# Supplementary material for: Identification of Genes Associated with Prognosis and Immunotherapy Prediction in Triple-Negative Breast Cancer via M1/M2 Macrophage Ratio
Source: Medicina (Kaunas). 2023 Jul 11;59(7):1285. doi: 10.3390/medicina59071285 (PMC10386704; doi:10.3390/medicina59071285)
Supplement: Supplementary file 1 [file medicina-59-01285-s001.zip › medicina-2414490-supplementary.pdf]

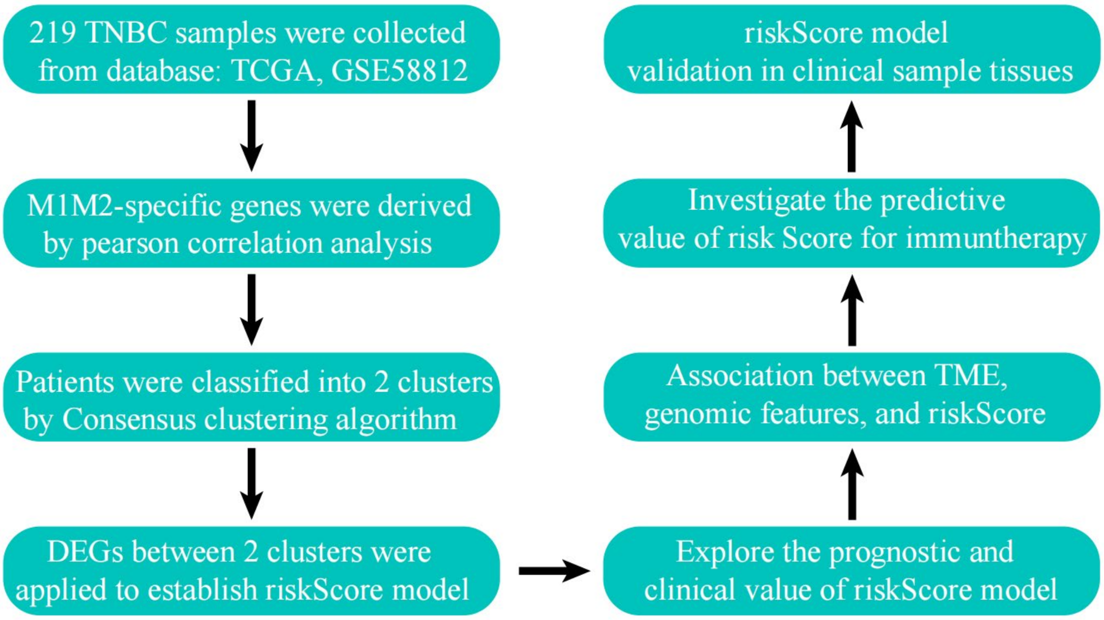


FIGURE S1| The flow chart of this study

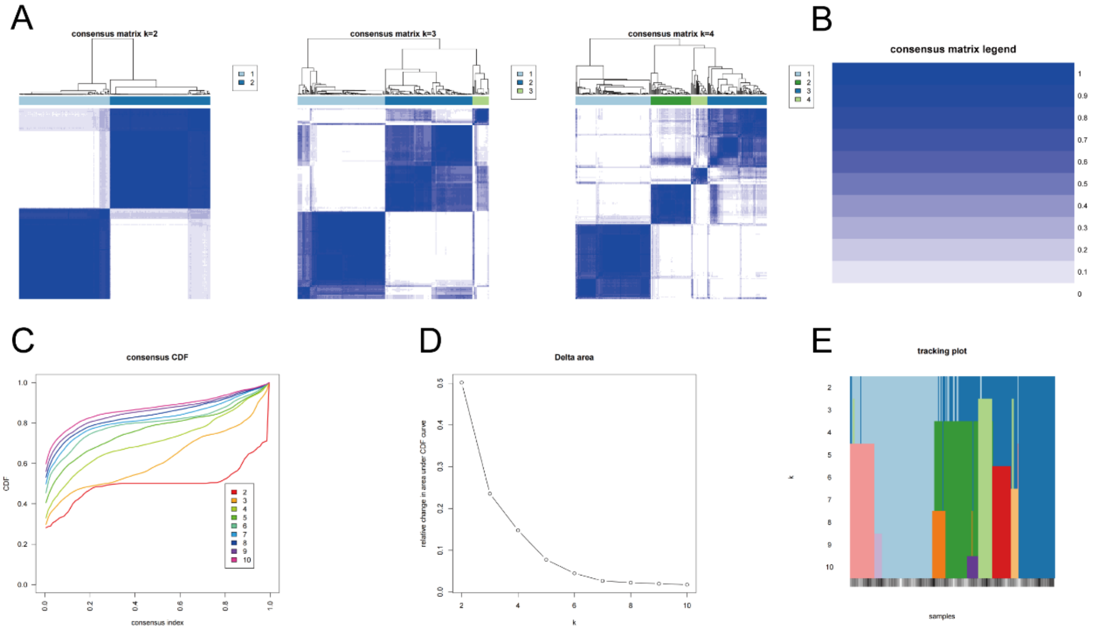


FIGURE S2| The clustering results were most stable when the number of individuals was 2 (K=2).

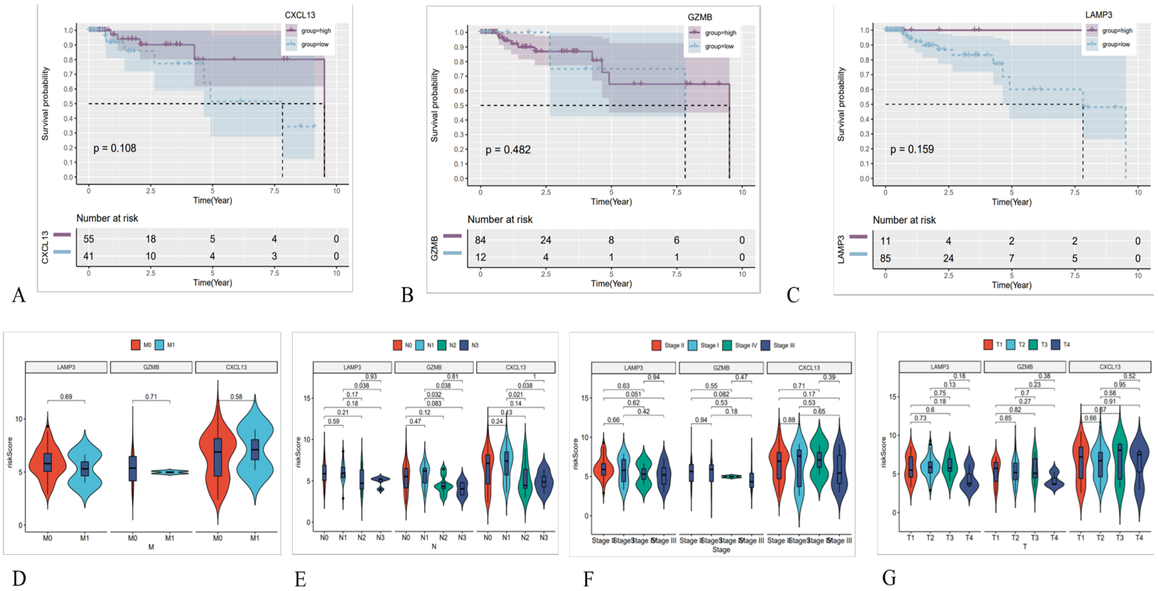


FIGURE S3| (A-C)The clinicopathological correlation of LAMP3, GZMB, and CXCL13 proteins, including survival analysis;(D-G) Expression levels across different T stages, N stages, and overall stages.

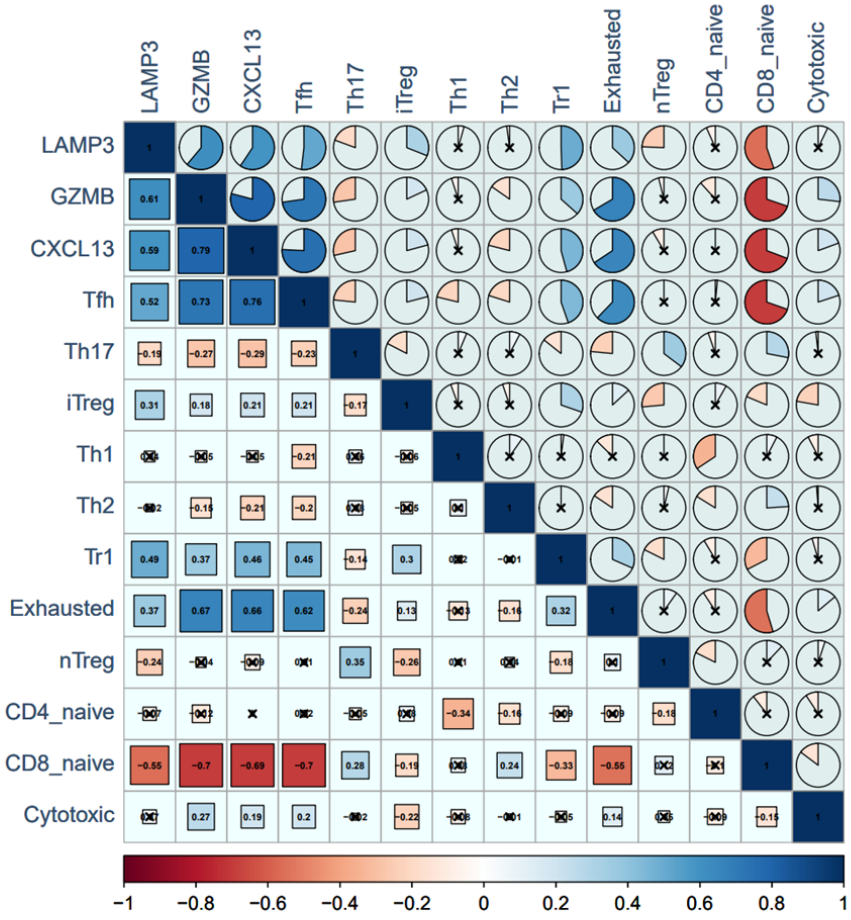


FIGURE S4|Spearman correlation analysis to investigate the relationship between the

expression levels of LAMP3, GZMB, and CXCL13 proteins and the cellular composition of the tumor microenvironment.

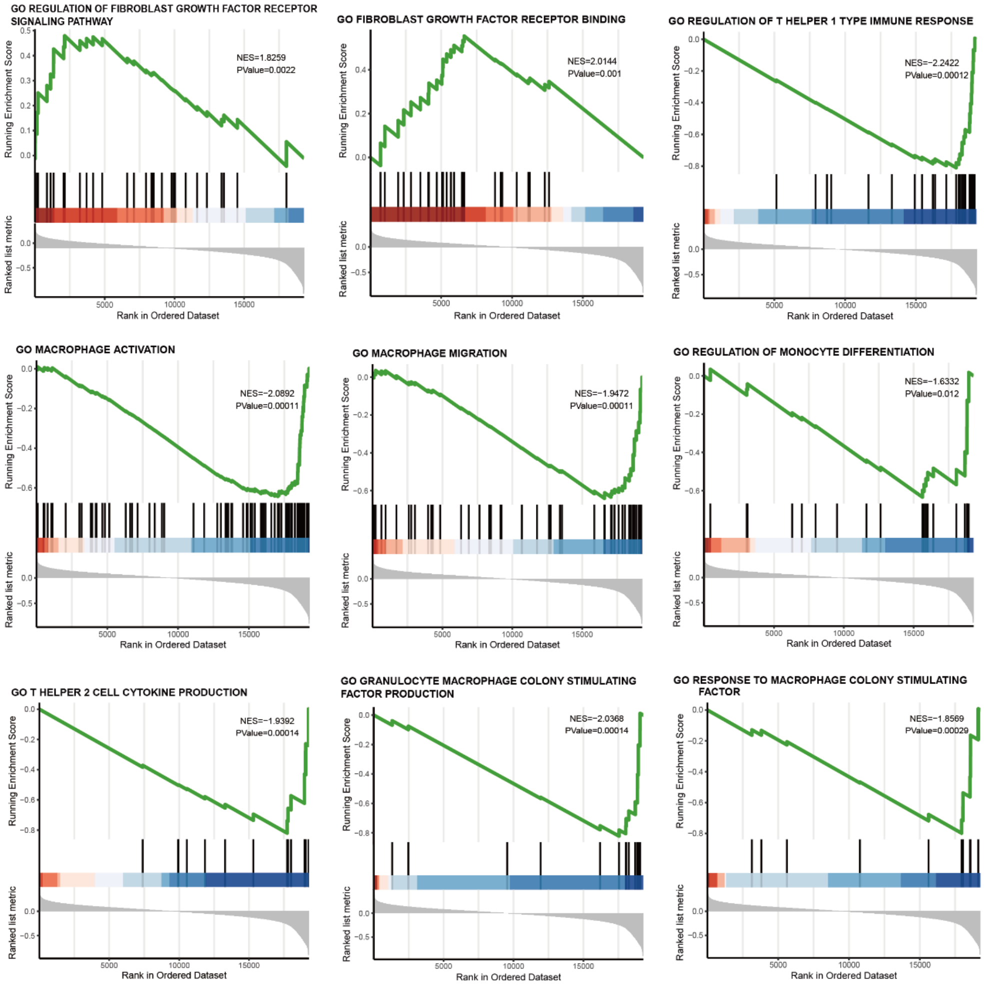


FIGURE S5| GSEA was applied to interpret the gene expression data of TCGA and GEO. The set of genes associated with macrophage activation and migration, GM-CSF production, and monocyte differentiation to TAM was relatively inactive in samples with higher riskScore.

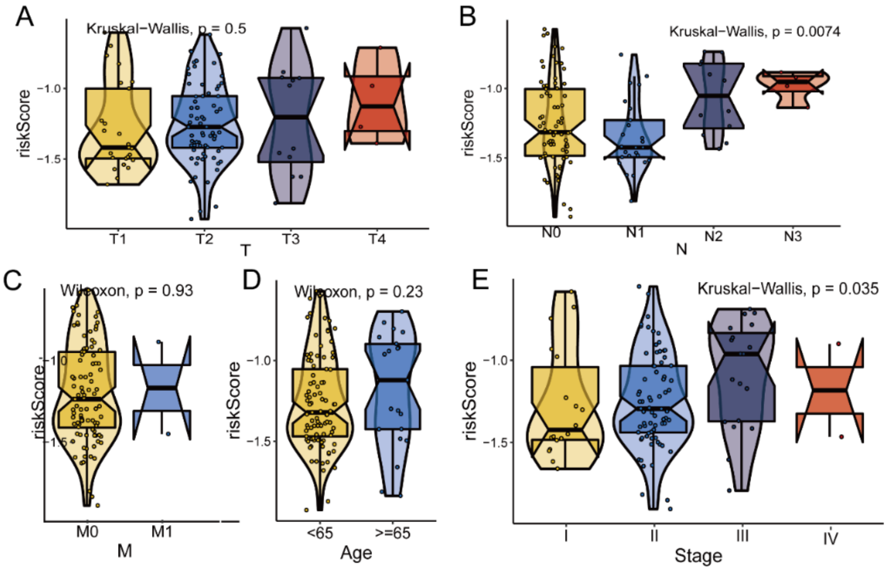


FIGURE S6| The significant differences in the AJCC staging of TNBC. the higher the N grade,

the higher the riskScore(S6B). However, the differences in age(S6D), metastasis(S6C) and T grade (S6A) on riskScore were not statistically significant.

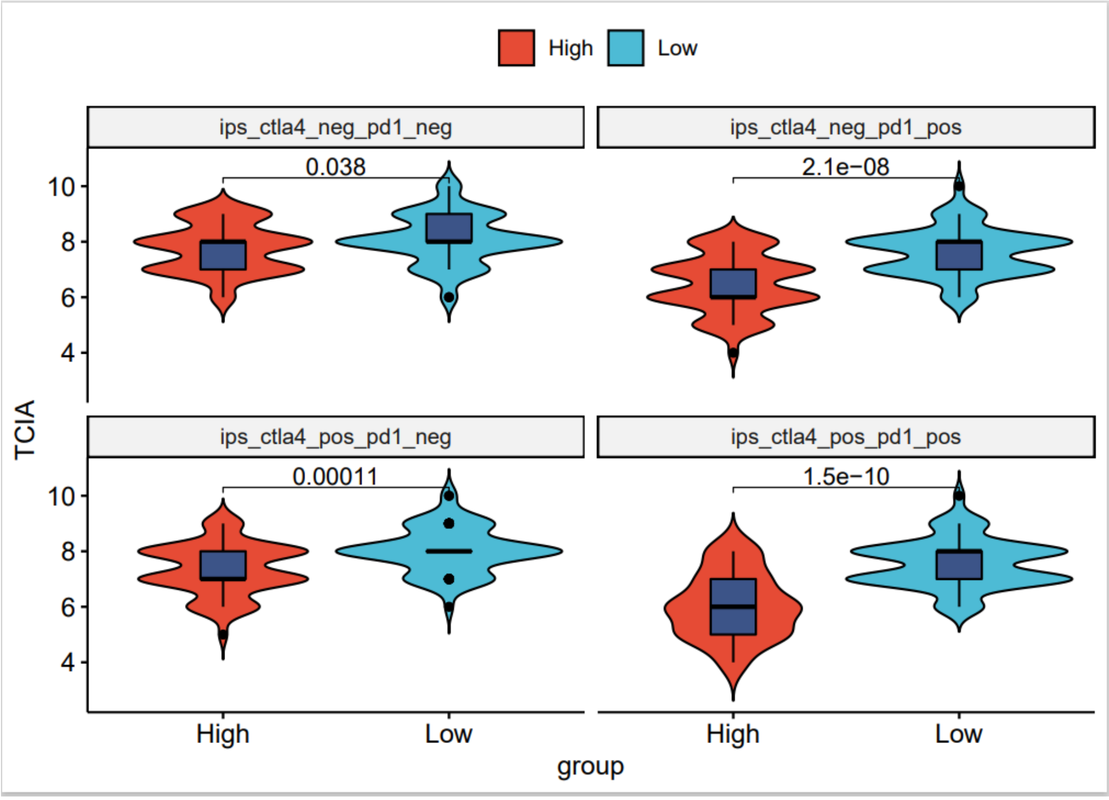


FIGURE S7| TCIA scores was evaluated for patients in the high and low riskScore groups.
